# Oliver Wendell Holmes

**Published:** 1887-10-01

**Authors:** 


### Oliver Wendell Holmes.

By Dr. Caterina.

When we know a man to be a charming poet, a novelist of unique quality, or an essayist of the first rank, we think of him with admiration, not wholly unmixed with envy; but when he is all three, and a professor of anatomy as well, we cannot help feeling that he is one of Nature's favoured children, to whom she has given more than his fair share of the good things of the intellectual life. Our ideas are all specialised now—large factories and the division of labour have done it; and while we respect a man for doing one thing well, we feel our mediocrity wronged when he succeeds equally, or almost equally well in two or three departments. But there are two consolations which we may take to heart; first, that if our more gifted fellow-mortal does more than his fair share of the world's intellectual work it atones in some degree for the stupidity and laziness which cause our tale of the bricks of thought to be so much smaller than it should be; secondly, that he uses his wealth in no miserly spirit, but invites us to partake of the meal which natural gifts, long, varied experience of life, and special training enable him to offer us, and is only the better pleased the more we show ourselves able to assimilate.

Besides, in thinking of the work of Dr. Oliver Wendell Holmes, we can perceive that many of the poems, and all the novels, and the delightful series of essays—the *Autocrat*, *Professor*, and *Poet at the Breakfast Table*—could not have been quite what they are if the author had not been "Doctor" Holmes. He admits that a friend talked of his "medicated novels," and we may, without malice, affix the same epithet to his other works, and say, for our further consolation, that *if* we had been destined to a professorial chair of some medical subject, we also might have produced similar volumes. Of course we know in our inmost hearts that we could have done nothing of the sort: that it is quite beyond our power to evolve the bright blossom of poetry or the ripe fruit of mellow, nutritive thought from the dry bones of anatomy, or even the less dessicated problems of physiology; but "your *if* is a great peacemaker," and reconciles us with ourselves.

And indeed it required the trained physician, as well as the literary artist, to give us such a portrait as that of Elsie Venner, whose character and fate are determined to such a great extent by inherited qualities; or the little crippled gentleman in the "Professor," with his strange, distorted views—strong, but not sound in mind as in body; and no one but a doctor could have known the combination of personal sympathy with professional interest which the Professor felt when he found out that the little gentleman was suffering from a disease so rare as displacement of the heart. Neither could anyone but a fellow-practitioner have ventured on that shrewd picture of the newly-qualified medical man, Dr. Benjamin Franklin, whom the poet consulted about a discolouration on his forehead. Dr. B. F., as he was familiarly called, had lately returned from Paris, and was possessed of ophthalmoscopes, and rhinoscopes, and otoscopes, and laryngoscopes, and stethoscopes, and thermometers, and spirometers, and dynamometers, and sphygmometers, and pleximeters, and probes, and probangs, and all sorts of frightful exploring contrivances, and scales to weigh you in, and tests, and balances, and pumps, and electro-magnets, and magneto-electric machines—in short, apparatus for doing everything but turn you inside out. Also, having seen as yet only hospital practice, he is most familiar with those ills which flesh is least commonly heir to, and has not yet realised that in general practice he will meet more frequently with measles than with elephantiasis, with indigestion than with empyema; so he talks in a learned and alarming way of *cutis ænea* and *Morbus Addisonii*—"a most rare and interesting disease." The poet gets frightened, but resolves to consult the landlady, Dr. B. F.'s mother. She diagnoses "bump," and prescribes vinegar and brown paper, under which treatment the *cutis ænea* speedily disappears.

Those of us who remember the days when our diplomas were still such new possessions that we could not help taking them out of their cases occasionally to read over their eccentric and hardly classical Latinity—much as the schoolboy likes to open all the blades of his pocket-knife at once—may also recall our first patients, whose cases we treated with the serious anxiety displayed by Dr. Benjamin Franklin; dreading meningitis at the mention of headache, and making all preparations for tracheotomy on hearing a complaint of sore-throat. But it is wonderful how quickly a little regular practice in, say, a colliery district or the poorer parts of a large town cures the young doctor of that little affectation, and makes him very glad that whooping-cough and rheumatism are only whooping-cough and measles, and not more "interesting" and deadly complaints. As the Master of Arts puts it in talking over Dr. B. F. with the poet, he begins practice *très bien chaussés*, as the French say, mightily well stored with professional knowledge. But when he begins walking round among his poor patients—he doesn't commonly start with millionaires—he finds that the new shoes of professional acquirements have got to be broken in, just like any other pair of new boots.

But besides these distinctly medical studies there are endless fragments of observation, epigrams which contain much solid wisdom in a witty form, which could come from no one who had not a good store of medical knowledge, as well as the general scientific habit of using his eyes. And if you think of it, there is no one who has such good opportunities for acquiring the material for essays as a medical man; and it is on his essays, his discursive talks on people and things in general, that Wendell Holmes's special fame will rest. The doctor sees human nature as it is, with all its artificial masks and integuments off, or at most worn so loosely as hardly to conceal the real form, either of soul or body. People don't make-believe to their doctor; they don't pretend to him to be better, or wiser, or richer than they are. If anybody in the world gets at the truth about people and their circumstances, it is he; there are more confessions poured into his ears than into many a priest's; and as a rule they are more genuine and spontaneous. I do not say that people always tell the absolute truth about either their mental or bodily troubles : that is impossible ; for, as the Autocrat points out, there are at least three personalities in every human body—1. The real man (known only to his Maker, says the observer) ; 2. The man's ideal self ; and 3. Other people's ideal of him—which two last often differ greatly from the first and from each other. But allowing for the fact that the patient is sure to be talking of number two and his doings, the doctor is, from the circumstances which occasion the interview, more likely than any other mortal to get a glimpse of number one.

Therefore, a doctor who has been in practice for fifteen or twenty years, as Dr. Holmes had been, before the *Autocrat of the Breakfast Table* was published, usually has a good deal to say that is worth listening to, though it chances that very few have either time or literary skill to set their information down in black and white. Of course there is required a certain maturity of mind that comes only from experience. Young observers generalise (in a very lordly style, too) from insufficient data ; they do so about flowers and insects, and other animals, and much more about the far more complicated problems of human nature ; but as human nature goes on unconsciously contradicting their pet theories, they outgrow that tendency and become humble. But you may trust a man of forty when he makes such a statement as this :—"It is better to lose a pint of blood from your veins than to have a nerve tapped. Nobody measures your nervous force as it runs away, nor bandages your brain-marrow after the operation." The Autocrat amplifies this dictum in speaking of what he calls the side-door to one's feelings—that door which leads directly to the secret chambers of the heart, the key of which should not be rashly given. For, he says, "if nature or accident has put one of these keys into the hands of a person who has the torturing instinct, I can only solemnly pronounce the words that justice utters over its doomed victim—'The Lord have mercy on your soul!' Some of those who enter by the side-door have a scale of your whole nervous system, and can play all the gamut of your sensibilities in semitones,—touching the naked nerve-pulps as a great pianist strikes the keys of his instrument. Married life is the school in which the most accomplished artists in this department are found. A delicate woman is the best instrument; she has such a magnificent compass of sensibilities! From the deep inward moan which follows pressure on the great nerves of sight, to the sharp cry as the filaments of taste are struck with a crashing sweep, is a range which no other instrument possesses. No stranger can get a great many notes of torture out of a human soul ; it takes one that knows it well—parent, child, brother, sister, intimate."

The tenderness towards women which this paragraph shows is characteristic of all Holmes's work. He has seen and noted the silent tragedies of women's lives, battles fought without a sound against the feebleness of woman's health, and the divinely implanted needs of women's nature. The prominent female characters in the three volumes of Essays are all working-women, and, to a certain extent, suffering women—the schoolmistress, brought up in luxury, bravely taking up unfamiliar work, and carrying it through under the burden of bereavement as well as of loss. Iris, with her rich artist-nature, her infinite capacities for love and sympathy, repressed under the stern rule of the model of all the virtues ; the young girl trying to earn her bread by literature. "It sounds well enough to say that she supports herself by her pen, but her lot is a trying one ; it repeats the doom of the Danaides. Imagine for a moment what it is to tell a tale that must flow on, flow ever without pausing. The lover miserable and happy this week, to begin miserable next week, and end as before. The villain scowling, plotting, punished ; to scowl, plot, and get punished again in our next ; an endless series of woes and blisses. Poor little body ! Poor little mind ! Poor little soul ! She is one of that great company of delicate, intelligent, emotional young creatures, who are waiting for some breath of heaven to fill their bosoms—love, the right of every woman ; religious emotion, sister of love, with the same passionate eyes, but cold, thin, bloodless hands—some enthusiasm of humanity or divinity ; and find that life offers them, instead, a seat on a wooden bench, a chain to fasten them to it, and a heavy oar to pull day and night. We read the Arabian tales, and pity the doomed lady who must amuse her lord and master from day to day, or have her head cut off ; how much better is a mouth without bread to fill it than no mouth at all to fill, because no bread. We have all around us a weary-eyed company of Scheherazades."

It is easy to pity these three—in fact, before the end of the volume there is a young man to pity each of them with that pity which is more than akin to love—identical with it. But less attractive women—the landlady, commonplace and garrulous, with those frequent plunges into the gulf of memory which send her grammatical construction into such a hopeless tangle ; and her shallow, sentimental daughter—get their due meed of appreciation and sympathy.

"Yes, my surface-thought laughs at you," says the Autocrat of the latter ; "you foolish, plain, over-dressed, mincing, cheaply-organised, self-saturated young person. But it is only my surface-thought that laughs. For that great procession of the Unloved, who not only wear the crown of thorns, but must hide it under the locks of brown or grey—hide it even from themselves—perhaps never know that they wear it, though it kills them—there is no depth of tenderness that Pity has not sounded. Somewhere—somewhere—love is in store for them ; the universe must not be allowed to fool them so cruelly. The great mystery of God's providence is the permitted crushing out of flowering instincts. Life is maintained by the respiration of oxygen and of sentiments. There comes a time when the souls of human beings, women perhaps, more even than men, begin to faint for the atmosphere of the affections they were made to breathe. What infinite pathos there is in the small, half-conscious artifices by which unattractive young persons seek to recommend themselves to the favour of those towards whom our dear sisters, the unloved, like the rest, are impelled by their God-given instincts !"

Still, in spite of his sympathy, it was with the schoolmistress that the Autocrat fell in love, and not with the landlady's daughter. But we can't wonder at that ; he has made us all love her too. In return, every woman reader of his confessions should love him for giving us the most exquisitely tender proposal of marriage in all literature. The Autocrat had drifted into the habit of escorting the schoolmistress to the scene of her labours. There were different ways that led to the school, and one of these, which the two liked best, they called the long path.

"I felt very weak indeed," says the Autocrat, in his account of the matter, "as we came opposite the head of this path on that morning. I think I tried to speak twice without making myself distinctly audible. At last I got out the question, 'Will you take the long path with me?' 'Certainly,' said the schoolmistress, 'with much pleasure.' 'Think,' I said, 'before you answer ; if you take the long path with me now I shall interpret it that we are to part no more.' The schoolmistress stepped back with a sudden movement, as if an arrow had struck her.

"One of the long granite blocks used as seats was hard by. 'Pray, sit down,' I said. 'No, no,' she answered softly, 'I will walk the long path with you.'"

(*To be continued.*)

